# Effects of Cardiopulmonary Rehabilitation on the Muscle Function of Children with Congenital Heart Disease: A Prospective Cohort Study

**DOI:** 10.3390/ijerph18115870

**Published:** 2021-05-30

**Authors:** Francisco José Ferrer-Sargues, Esteban Peiró-Molina, Maria Àngels Cebrià i Iranzo, José Ignacio Carrasco Moreno, Ana Cano-Sánchez, María Isabel Vázquez-Arce, Beatriz Insa Albert, Pablo Salvador-Coloma

**Affiliations:** 1 Department of Physiotherapy, Universidad Cardenal Herrera-CEU, CEU Universities, Alfara del Patriarca, 46113 Valencia, Spain; franciscojose.ferrer@uchceu.es (F.J.F.-S.); pablo.salvador@uchceu.es (P.S.-C.); 2 Pediatric Cardiology Section, Hospital Universitari i Politècnic La Fe, 46026 Valencia, Spain; Estebanpeiromolina@gmail.com (E.P.-M.); carrasco_jim@hotmail.com (J.I.C.M.); acano20081976@gmail.com (A.C.-S.); beatriz.insa@gmail.com (B.I.A.); 3 Regenerative Medicine and Heart Transplantation Unit, Instituto de Investigación Sanitaria La Fe, 46026 Valencia, Spain; 4 Department of Physiotherapy, Universitat de València, 46010 Valencia, Spain; 5 Rehabilitation and Physical Medicine Service, Hospital Universitari i Politècnic La Fe, 46026 Valencia, Spain; isabel.vazquez.arce@gmail.com; 6 Faculty of Medicine and Health Sciences, Universidad San Vicente Mártir, 46001 Valencia, Spain

**Keywords:** congenital abnormalities, cardiac rehabilitation, pediatric, resistance training, muscle strength, exercise

## Abstract

Critical medical and surgical advances have led to a shift in the care and management of children with congenital heart disease (CHD). These patients present with muscle deconditioning, which negatively influences their response to exercise, functional capacities, and quality of life. This study evaluates the influence of a cardiopulmonary rehabilitation program (CPRP) on the function of peripheral musculature of children with CHD. A single-center prospective cohort study was designed. Fifteen CHD subjects, between 12 and 16 years of age, with reduced aerobic capacity on a cardiopulmonary exercise test, were included in a three-month, 24-session CPRP. Measurements of the subjects’ handgrip strength, biceps brachii and quadriceps femoris strength, and triceps surae fatigue process were collected at the beginning of the program, after completion, and six months after the end of the intervention. A substantial and statistically significant improvement was observed in the subjects’ handgrip strength (kg) (*p* < 0.001), biceps brachii and quadriceps femoris strength (N) (*p* < 0.001), as well as triceps surae fatigue process (repetitions) (*p* = 0.018), with a maintenance of the results six months after the intervention. These results suggest that a CPRP could potentially improve the peripheral muscle function of children with CHD. Additional research is needed to confirm and expand on this hypothesis.

## 1. Introduction

Congenital heart disease (CHD) represents the most common cause of congenital malformations, with an estimated incidence bordering on 8–10‰ of live births [1], and it has a significant impact on health indicators and the sanitary economy worldwide [2]. Over the course of the last few decades, important advances in surgical techniques and medical management have prominently increased survival, allowing CHD patients to live through adulthood [3]. After overcoming these hurdles in patient survival, researchers have been increasingly shifting the focus of their studies and interventions from avoiding deaths to attaining a greater health-related quality of life (HRQoL) [4] for patients.

CHD patients have a reduced exercise capacity when compared to the general population, and some studies have associated this reduction with the hemodynamic repercussions of the cardiac defects, factors related to cardiac surgery, chronotropic incompetence, and underlying lung disease [5]. However, there is previous evidence that exercise capacity is not determined by the cardiac variables in isolation but depends on a complex interplay between cardiopulmonary and muscular factors [6].

Children with CHD usually present with muscle deconditioning, myopathy, and muscular weakness [7]. It has been reported that the majority of these patients do not reach the current physical activity recommendations outlined by the World Health Organization, consisting of 60 min per day of moderate to vigorous physical activity [8], Furthermore, some of them suffer from imposed restrictions on participation in physical activities [9], which could negatively influence their functional capacities, exercise response, and quality of life [10].

In the past several years, resistance training has proven to be a safe and effective method of conditioning for healthy children, supported by the American Academy of Pediatrics [11], the National Strength and Conditioning Association [12], and the American College of Sports Medicine (ACSM) [13]. These recommendations can be adapted for children with CHD with appropriately designed and competently supervised resistance training programs [14].

This study aims to evaluate the effect of a systematic cardiopulmonary rehabilitation program (CPRP) including strength-resistance training on the peripheral muscle function of children with congenital heart disease.

## 2. Materials and Methods

### 2.1. Study Design

A single-center prospective cohort study was designed and conducted in compliance with the Good Clinical Practices protocol and the Declaration of Helsinki principles. It was approved by the Health Research Institute Hospital La Fe (Valencia, Spain) Ethics Committee on 4 December 2017, with the registration number 2017/0506. The patient information sheet was explained and all subjects and their legal guardians gave their informed consent for inclusion before they participated in the study.

### 2.2. Participants

All the participants were recruited from the Pediatric Cardiology Section of the Hospital Universitari i Politècnic La Fe (Valencia, Spain) between December 2017 and January 2020 by screening all patients scheduled for cardiopulmonary exercise testing in the exercise physiology laboratory.

Inclusion criteria were defined as: (a) age between 10 and 16 years; (b) height greater than 135 cm; (c) the presence of a significant congenital heart abnormality based on the European Society of Cardiology Guidelines of the Management of Adult Congenital Heart Disease [15]; (d) an abnormal exercise capacity, defined as a peak oxygen consumption of less than 80% of predicted values [16]; (e) willingness to be part of the study and participation commitment from the patients and their parents or legal tutors; (f) a signature on the informed consent form after being given thorough program and study information. 

We excluded any patients presenting (a) a personal history of documented life-threatening arrhythmias; (b) the inability or contraindication to perform the required physical activity; (c) a significant depression of left or right ventricle function; (d) hypotensive response to exercise in Cardiopulmonary Exercise Testing (CPET). 

All measurements, evaluations, and interventions performed in the context of the present study were performed in a safe environment, which ensured the availability of resuscitation material and devices. The subjects’ vitals and continuous ECG (Nuubo^®^ wearable ECG technology, Nuubo, 28043 Madrid, Spain) signals were monitored by a pediatric cardiologist.

### 2.3. Measurements

#### 2.3.1. Anthropometric Characteristics

Anthropometric measurements were collected from all participants, including their height (cm) and weight (kg). BMI (kg/m^2^) was calculated by dividing each participant’s weight by the square of their height in meters. Standard deviation (SD) scores were calculated for weight, height, and BMI according to the Spanish population standards recently published by Carrascosa et al. [17].

#### 2.3.2. Muscle Function

Each participant’s handgrip strength (kg) was evaluated in both hands using a Jamar Plus+^®^ device (Patterson Medical, Sammons Preston, Bolingbrook, IL, USA) [18]. The results were compared to the reference values in the owner’s manual, available for ages 6–75 years. The biceps brachii and quadriceps femoris strengths (Newton, N) were evaluated on the arms and legs using a dynamometry Lafayette Manual Muscle Tester device (Lafayette, IN, USA) [19]. The measuring technique is described by Bohannon et al. [20]. Finally, the fatigue process of the triceps surae was evaluated using the single-heel rise test, with a maximum of 25 repetitions [21], taking this value as a reference because this is the average number of repetitions performed by a healthy member of the population [22]. All the strength measurement techniques were selected according to their validity, reliability, and ease of use for a pediatric population [23] with congenital heart disease [24].

To minimize each subject’s training and motivation interference, a careful explanation of the procedure was carried out, the subjects were vigorously encouraged, and each measurement was repeated until three acceptable and reproducible values (with a difference of <10%) were registered, with a one-minute rest between them. The highest measurement was then registered. 

All the measurements were collected at the beginning of the program (Before), after completion (After), and six months after the conclusion of the last session (After 6 m). All tests were carried out at the same location in the hospital and at the same time of day. Children were given some instructions, including: restricting food for two h before the tests, not practicing sports on the same day, and mandatory reporting of any musculoskeletal injuries sustained in the last week.

### 2.4. Intervention

All the participants were included in a pediatric CPRP named the IMPROVE project (Initiative for Monitored Pediatric cardiac Rehabilitation Oriented by cardiopulmonary Exercise testing). The IMPROVE intervention was designed by following the American College of Sports Medicine (ACSM) Guidelines for exercise prescription, considering the FITT (Frequency, Intensity, Type, and Time) principles for cardiac patients, and adjusting them for the pediatric population.

Frequency was set to two times a week for a total of 24 sessions. Sessions lasted 70 min, including endurance and strength-resistance training. Intensity was defined by the subject’s CPET, initially aiming for a heart rate (HR) near the first ventilatory threshold (VT1) HR, and displacing this target frequency progressively throughout the program towards the secondary ventilatory threshold (VT2) HR or a maximal HR of 75% of their peak HR in cases where the VT2 was not available. 

The patient’s heart rate (bpm), blood pressure (mmHg), peripheral oxygen saturation (SpO2, %), and the perceived exertion using the Borg CR-10 Scale were recorded at the beginning and the end of each session and after the endurance and resistance training phases. The training was led by two experienced physiotherapists and supervised by a pediatric cardiologist.

The sessions were structured as follows: (a) Warm-up phase (5 min): this included diaphragmatic breathing, articular mobility exercises, and a light walk. (b) Endurance-training phase (20 min): exercise was carried out in a continuous modality using a treadmill (Magna Pro RC, BH Fitness, Madrid, Spain), and a static bicycle (BH Rhyno Max H491, BH Fitness, Madrid, Spain), and included two min of warm-up and another two min of cool-down. The first eight sessions were performed in a uniform continuous modality, adjusting the intensity to the subject’s VT1 HR. In sessions 09–16, the load was increased progressively up to the VT2. The last eight sessions included rhythm modulations, switching to varying continuous training, which oscillated between the VT1 and the VT2 HR [25]. (c) Resistance-training phase (20 min): during the first eight sessions, the subjects completed three series with four analytical exercises, working out especially eight muscle groups (the deltoids, biceps brachii, triceps brachii, abdominals, trunk extensors, quadriceps, hamstrings, and calves) [10,26]. The subjects made 10–15 repetitions of each exercise, with a 20 s rest. The training was carried out with light and medium resistance bands. In the following eight sessions, we emphasized exercises that included neuromuscular control using gymnastics equipment such as dumbbells, bosu, medicine balls, steps, and Pilates balls, as well as doing plyometric workouts. These functional routines included three series with four exercises in each one. The subjects completed 10–15 repetitions or 40 s work for each exercise, with a 20 s rest. During the last eight sessions, multi-circuits and adaptive non-competitive sports were trained, in addition to exercises related to daily living activities. The routines were performed in groups. To complement the training and to provide the patient with a recreational component, the last sessions sporadically included virtual reality games. (d) Respiratory-training phase (20 min): as a final phase of muscular training, a respiratory musculature workout was performed using an Inspiratory Muscle Trainer Threshold (Respironics Respiratory Drug Delivery, Chichester, UK), working at least 30% of the subjects’ Maximum Static Inspiratory Pressure [27]. (e) Cool-down phase (5 min): this included a light walk and body stretching.

### 2.5. Statistical Analyses

Data treatment and visualization was performed using the Python open-source libraries including Numpy©, Pandas©, Matplotlib©, Seaborn©, Scypy© and StatsModel©. The distribution of quantitative variables was tested for normality before inferential analysis by performing the Shapiro–Wilk, D’Agostino K^2, and Anderson–Darling tests. The bivariate association was investigated using a paired t-test for the normally distributed variables and a Wilcoxon signed-rank test for the non-normally distributed variables. Bonferroni correction was applied to account for multiple measurement comparisons and potential alpha error. Data are presented as mean values ± SD. A *p*-value < 0.05 was considered statistically significant. The sample size calculation for paired mean differences was calculated assuming a level of significance of 0.05, a statistical power of 70%, and a moderate effect size of 0.6 in favor of handgrip strength improvement, resulting in a minimum sample size of 14 patients.

## 3. Results

### 3.1. Population

All 353 children tested at the exercise physiology laboratory throughout the study duration were evaluated for eligibility for inclusion. Twenty-eight fulfilled the inclusion criteria. Of these, 13 subjects or their legal guardians declined to participate in the study. The main reasons given when rejecting participation were geographical limitations and the time-consuming exigencies of the program, respectively. Amongst the patients that fulfilled clinical criteria, there were no significant differences between the ones that accepted and rejected participation in terms of gender, age, or anthropometric characteristics. There were also no differences in our sample between dominant and non-dominant limbs, as was also reported in the systematic review published by Bohannon et al. [28]. 

A total of 15 subjects were enrolled in the study, with a mean age of 14.4 (Range 12.4–15.7), and a gender distribution of 60% male–40% female. Patients’ diagnoses were Tetralogy of Fallot (*n* = 6), heart transplantation derived from CHD (*n* = 3), d-transposition of great arteries corrected with an arterial switch (*n* = 2), pulmonary atresia with intact ventricular septum (*n* = 1), pulmonary atresia with ventricular septal defect (VSD) (*n* = 1), repaired VSD (*n* = 1), and repaired Taussig–Bing anomaly (*n* = 1). The transplanted subjects’ primary diseases were tricuspid atresia with Fontan surgery, aortic coarctation with VSD, and non-compacted cardiomyopathy with severe ventricular dysfunction. 

Regarding functional capacity, 12 subjects were in New York Heart Association (NYHA) class I, and 3 subjects were classified as NYHA class II at the beginning of the study. 

The demographic and anthropometric features of the study population are described in Table 1. No significant differences were observed between boys and girls.

### 3.2. Program Adherence and Safety

All the patients completed the study’s goal of performing more than 75% of the programmed training sessions. On average, each patient missed three training sessions (12%, range, 1–5 sessions). No adverse events were reported during rehabilitation, except for minor muscle stiffness in the first week of training. The ECGs showed no significant arrhythmias, only registering infrequent and non-perceived monotopic ventricular ectopy in one patient. 

### 3.3. Muscle Function

All the participants successfully performed the muscle function measurements at all programmed timepoints without any incidents. All the patients were right-handed, and no significant differences were noticed in the strength improvement between the dominant and non-dominant sides of all studied muscle groups. Although the baseline muscle strength was generally higher in dominant extremities, this difference was statistically non-significant in all muscle groups. A significant increase in strength after the training program was observed in all measured muscle groups for both dominant and non-dominant sides. Muscle function measurement results from before and after the program are summarized in Table 2.

**Figure 1:**
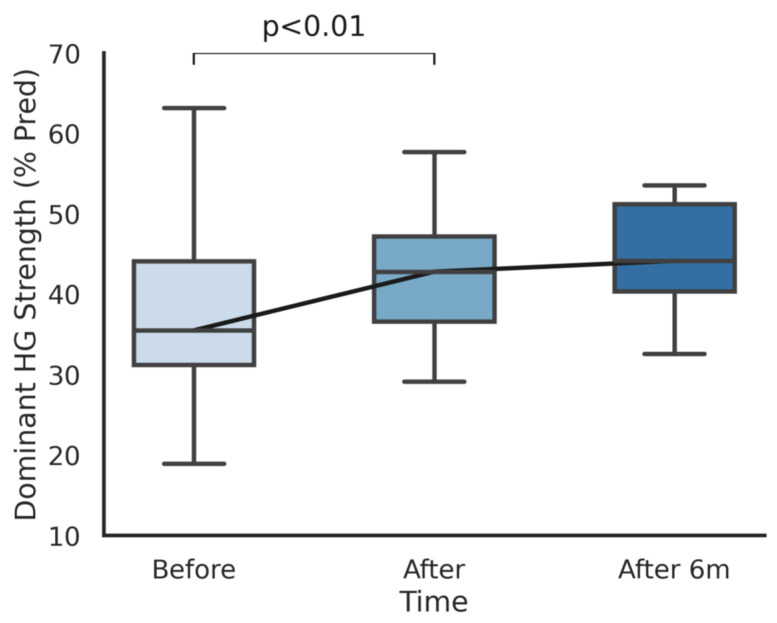
The percentage of predicted handgrip strength in the dominant hand before, after, and 6 months after the completion of the cardiopulmonary rehabilitation program.

**Figure 2:**
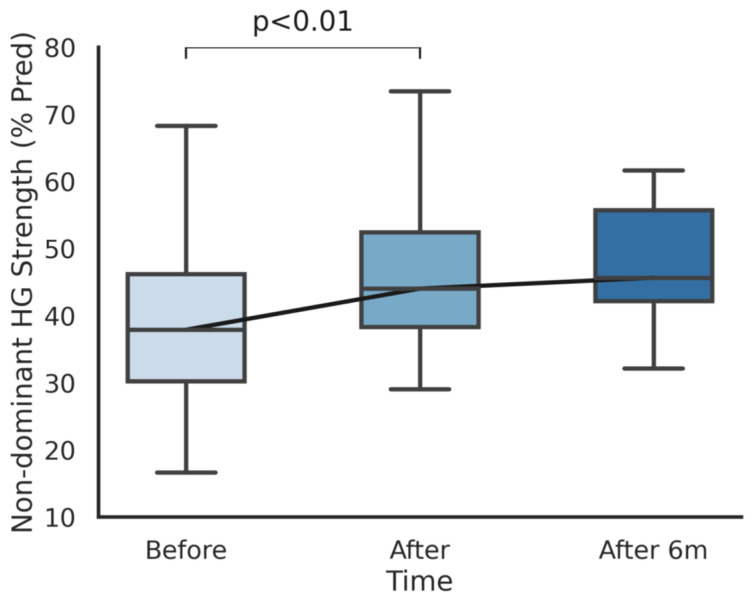
The percentage of predicted handgrip strength in the non-dominant hand before, after, and 6 months after the completion of the cardiopulmonary rehabilitation program.

Handgrip strength increased by an average of 4.1/4.7 kg (17/21%) in dominant/non dominant hands after training (*p* < 0.001). In order to normalize values and minimize the effect of mere growth, the availability of reference values for our population allowed us to compare the percentage of predicted handgrip values recalculated with up-to-date height and weight measurements. We reported an increase in the percentage of predicted handgrip values from 37% to 44% (*p* < 0.001) for the dominant hand and from 39% to 47% (*p* < 0.001) for the non-dominant hand. The improvements in handgrip strength for both the dominant and non-dominant sides are shown in Figure 1 and Figure 2, respectively.

An increase in biceps brachii strength was observed after training in both the dominant (118 to 140 N, *p* < 0.001) and non-dominant arms (117 to 132 N, *p* < 0.001). Similarly, we evidenced an increase in quadriceps femoris strength in the dominant (161 to 204 N, *p* < 0.001) and non-dominant (153 to 185 N, *p* < 0.001) legs. The single-heel rise test performance rose from an average of 10.4 to 16 repetitions (*p* = 0.018) in the dominant leg and from 9.2 to 16.6 in the non-dominant extremity (*p* < 0.001).

The follow-up measurements revealed, interestingly, that six months after ceasing the CPRP there were no statistically significant changes in any of the tests performed, rendering the changes produced by sheer growth non-significant, and supporting the hypothesis that the effects observed immediately after the intervention could be related to it. The results of the follow-up measurements and their comparison with the values at the time of completion of the program can be examined in Table 3.

## 4. Discussion

This study observed a general baseline impairment of peripheral muscle function in children with CHD, showed an improvement in hand, arm, and leg muscle strength after a 24-session CPRP. The strength gains have been maintained after a period of 6 months following the intervention.

Muscle function alteration in relation to CHD has been a topic of infrequent but fruitful study over the last two decades. Even though the muscle and bone structure of patients with CHD has been reported to be similar to that of healthy subjects when normalized by height [29], muscular weakness has been repeatedly pointed out in the literature. A study carried out in adolescents and young adults with CHD by Fricke et al. revealed decreased muscle power when compared to the general population [30]. Kröönström et al. published a study showing a handgrip strength of 90%/87% in males/females with CHD when compared to healthy people [31]. Handgrip strength was the only parameter that could be compared to predicted values based on gender and age, since no reference values were found for the rest of the measured variables in the pediatric population [18]. According to these reference values, our results suggest a notably marked decrease in baseline handgrip strength in children with CHD when compared to those values obtained from healthy historical controls.

Muscle function is a predictor of long-term survival, and both muscular strength and endurance have been directly related to exercise tolerance [32]. A CPRP including aerobic and resistance training could be a good intervention for CHD children, as the increased exercise capacity observed after a period of training has been attributed more to the peripheral than to the central adaptations [33]. Even though some studies have measured the effect of a CPRP in children with CHD, very few have evaluated muscle function. Our group previously evaluated the benefits of cardiac training on respiratory muscle strength in this population, finding an improvement in the Maximum Static Inspiratory Pressure and the distance achieved in a 6 min walking test [27]. Other groups such as Moalla et al. have observed a significant increase in the maximal voluntary contraction, despite being a home-based intervention [34]. A study by Brassard et al. reported no significant improvement in the maximal voluntary contraction or time to fatigue [35], though these results could be due to a reduced sample size (*n* = 4). Our results show a considerable and statistically significant improvement in all measurements after the completion of a cardiopulmonary training program, supporting the evidence of some of the previously cited studies.

Significant heterogeneity exists in CPRP methodologies, favoring aerobic training programs over strength-resistance workouts [14,36]. Furthermore, these programs do not clearly describe any progression of the exercises during the program, except for Moalla et al. [34], who recommend continuously adjusting training intensity to improve cardiorespiratory function and muscle performance. A highlight of our study was the division of both the endurance and strength-resistance training programs into three incremental phases, with eight sessions each. The first phase performs analytical workouts of the main muscle groups and an assessment of the subject’s skills and deficiencies. Throughout the second phase, we performed strength exercises focused on neuromuscular control, since it has been proven that they promote the quality and efficiency of movement, in addition to preventing injuries caused by lack of muscle control [37]. The last sessions included non-competitive recreational games in order to promote functional training and emulate real-life activities. This workout was also intertwined with virtual reality games that have demonstrated positive hemodynamic effects in patients with coronary disease [38] and children with cystic fibrosis [39].

In addition to central and peripheral factors, the impaired physical activity in these patients could also be the result of parental and environmental overprotection [40]. Parents’ perception of their children constitutes an interesting discussion topic involving healthcare specialists. Even though parents may consider themselves to be those most responsible for their children’s wellbeing, they often feel insufficiently informed by health professionals. From this perspective, rehabilitation should have the ultimate goal of providing children with enough knowledge and confidence to catalyze their growth and maturation towards adulthood, and increase their perceived HRQoL [41]. In our study, we witnessed an extraordinarily favorable predisposition in children and their families towards the training program, and a very high completion rate.

The present study possesses limitations that could influence its interpretation. Firstly, the total sample size of the study is small, as described in most pediatric rehabilitation systematic reviews [14]. This phenomenon is due to the intensive time and resource requirements of the CPRP. We considered it advisable to reduce the number of participants per group in order to increase safety and training quality. A second limitation is diagnosis heterogeneity, which could affect the extrapolation of the results to the whole population of children with CHD. This is caused by the variability of subjects with CHD who are susceptible to cardiac rehabilitation. Additionally, the lack of a control group constitutes a limitation. This design decision was made due to the scarce number of CHD patients and the elevated time and resource costs for the control families. To counterbalance this issue, the potential confounding factors were discussed initially and the paramount confounding factors were identified as the children’s growth and its effect on their training. To reduce the impacts of the first factor, we used predicted values instead of absolute values when possible and compared the improvements observed during the three months of training with the evolution of the same variable in the six-month period after the end of the program, giving us an approximate estimate of the effect of natural growth. To minimize the impacts of the second confounding factor, the same evaluator thoroughly trained the subjects prior to every measurement and always aimed for consistency in the data acquisition process. Lastly, it could be interpreted that the six-month follow-up results could be affected by the amount of physical activity that each child had participated in over that period. To minimize this potential bias, we created a dossier containing aerobic and strength-resistance training exercises that all the subjects received at the end of the program, and we encouraged them to join a gym or practice non-competitive recreational sport in order to encourage them to stay as active as possible.

## 5. Conclusions

In conclusion, our results found an increase in peripheral muscle function after a three month 24-session CPRP in children with CHD. This improvement persisted 6 months after the completion of the program. These results provide objective and specific information that could help rehabilitators, cardiologists, and physiotherapists to plan, design, and execute strategies to improve the functional capacities of children with congenital heart disease through exercise and potentially impact their HRQoL.

Our results expand on prior research that points to a progression in intensity as a key factor in the improvement of muscle function. A design including strength-resistance, aerobic, and respiratory training may be a good starting point for future studies. These studies could potentially confirm our results and expand on this particular topic, generating a robust foundation of evidence in order to improve our practice and medical advice and work towards a healthier lifestyle for CHD children.

## Figures and Tables

**Table 1:** Demographic and anthropometric characteristics of the study population (*n* = 15).

	Total (*n*= 15) Mean ± SD (Range)	Boys (*n*= 9) Mean ± SD (Range)	Girls (*n*= 6) Mean ± SD (Range)	*p*-Value
Age (years)	14.4 ± 1.1 (12.4–15.7)	14.4 ± 1.3 (12.4–15.7)	14.5 ± 0.9 (13.3–15.8)	0.43
Height (cm)	161.9 ± 9.9 (143–182)	164.9 ± 10.7 (143–182)	157.4 ± 7.3 (145–165)	0.05
Weight (kg)	52.8 ± 12.5 (33–74.2)	55.5 ± 12.9 (41.3–74.2)	48.9 ± 11.9 (33–63)	0.29
BMI (kg/m^2^)	20 ± 3.5 (14.8–25.4)	20.3 ± 3.6 (14.8–25.4)	19.5 ± 3.8 (15.7–24.3)	0.11

Abbreviations: BMI = Body Mass Index; SD = standard deviation.

**Table 2:** Comparison of muscle function before and after training (*n* = 15).

	Before	After	Change (%)	Mean Difference	*p*-Value
Dom Hand grip (kg)	24 ± 8.6	28.1 ± 9.2	17	4.1	<0.001
N-Dom Hand grip (kg)	21.9 ± 7.9	26.6 ± 9.2	21.4	4.7	<0.001
Dom Biceps brachii (N)	118.1 ± 26.3	139.5 ± 37.8	18.1	21.4	<0.001
N-Dom Biceps brachii (N)	116.7 ± 27.2	132.4 ± 26.4	13.4	15.7	<0.001
Dom Quadriceps fem (N)	160.5 ± 40.8	204 ± 48.7	27.4	44	<0.001
N-Dom Quadriceps fem (N)	152.8 ± 48.3	184.9 ± 44.1	21	32.1	<0.001
Dom Single-heel rise (rep)	10.4 ± 7.5	16 ± 8.3	53.8	5.6	0.018
N-Dom Single-heel rise (rep)	9.2 ± 6.3	16.6 ± 8.1	80.4	7.4	<0.001

Abbreviations: fem = femoris; Dom = Dominant arm/leg; N-Dom = Non-dominant arm/leg; rep = repetitions.

**Table 3:** Comparison of muscle function after training and at the 6-month follow-up (*n* = 15).

	After	After 6 m	Change (%)	Mean Difference	*p*-Value
Dom Hand grip (kg)	28.1 ± 9.2	29.7 ± 10	5.7	1.6	ns
N-Dom Hand grip (kg)	26.6 ± 9.2	27.8 ± 8.8	4.5	1.2	ns
Dom Biceps brachii (N)	139.5 ± 37.8	145.5 ± 47.1	4.3	6	ns
N-Dom Biceps brachii (N)	132.4 ± 26.4	138.5 ± 43.5	4.6	6.1	ns
Dom Quadriceps fem (N)	204 ± 48.7	189.5 ± 49.4	−9.2	−14.5	ns
N-Dom Quadriceps fem (N)	184.9 ± 44.1	188.6 ± 49.2	2	3.7	ns
Dom Single-heel rise (rep)	16 ± 8.3	18 ± 7	12.5	2	ns
N-Dom Single-heel rise (rep)	16.6 ± 8.1	20.7 ± 7.1	29.3	4.1	ns

Abbreviations: ns = non-significant (>0.05); fem = femoris; Dom = Dominant arm/leg; N-Dom = Non-dominant arm/leg; rep = repetitions.

## Data Availability

Not appliable.
